# Mechanical Behavior of Fine Recycled Concrete Aggregate Concrete with the Mineral Admixtures

**DOI:** 10.3390/ma13102264

**Published:** 2020-05-14

**Authors:** Minkwan Ju, Jae-Gwon Jeong, Martin Palou, Kyoungsoo Park

**Affiliations:** 1 Department of Civil and Environmental Engineering, Yonsei University, Seoul 03722, Korea; j_dean21@naver.com (M.J.); k-park@yonsei.ac.kr (K.P.); 2 Global R&D Center, SQ Engineering, Seoul 05818, Korea; 3 Construction and Architecture, Slovakia Academy of Science, 84503 Bratislava, Slovakia; martin.palou@savba.sk

**Keywords:** fine recycled concrete aggregate, fly ash, ground-granulated blast-furnace slag, silica fume, mechanical behavior, effective dosage

## Abstract

The paper describes the mechanical behavior of fine recycled concrete aggregate (FRCA) concrete according to the mineral admixtures. Three types of the mineral admixtures, i.e., fly ash (FA), ground-granulated blast-furnace slag (GGBS), and silica fume (SF), are used and the replacement ratios of FRCA are 50% and 100%. The dosages of the admixtures of FA, GGBS, and SF are determined with the normal dosage (30%, 40%, and 5.0%, respectively) based on the ACI committee reports (No. 232, 233, and 234) and half-normal dosage. The mechanical performance is investigated with the compressive and splitting tensile strength, and elastic modulus. Additionally, the total porosity is measured in natural fine aggregate (NFA) and FRCA 100% replaced specimens by mercury intrusion porosimetry (MIP) for investigating the relationship with the compressive strength. Based on the experimental test results, the mineral admixtures improve the mechanical performance of FRCA concrete. The effective dosages of FA, GGBS, and SF for FRCA concrete are investigated according to the replacement ratio of the FRCA. In particular, FRCA 100% replaced concrete may be possible to be used for the structural concrete members with the specific dosage of the mineral admixtures. The prediction of the splitting tensile strength and the elastic modulus by the codes or previous formulas exhibits underestimated and overestimated results, respectively. The relationship between the total porosity and the compressive strength of the FRCA concrete should be modified with more experimental tests.

## 1. Introduction

The depletion of natural aggregates in construction has been a significant reason to begin the use of substitutes, such as artificial aggregates, wastes, and recycled products. One of the promising materials is the recycled concrete aggregate (RCA) from demolition of the concrete members [1,2]. This recycling process is familiar and practical achievement which can friendly feedback them in the construction field. However, the old cement paste on the surface of RCA can bring the adverse effects on material property. For example, the relatively high porosity in the old cement paste leads to poor characteristics of the interfacial transition zone (ITZ) [3,4].

The FRCA concrete is more difficult to incorporate into the structural concrete than coarse recycled concrete aggregate (CRCA) concrete, mainly due to FRCA’s water absorption that result in workability issues with fresh concrete [5]. Many previous studies have presented water absorptions of FRCA in the range of 6–13%, corresponding to the compressive strength reduction of 4–36% [6,7,8,9,10,11]. Whereas, for the relatively good quality of FRCA with 5.4% of water absorption, the strength reduction of the compressive and splitting tensile strength showed 26% and 30%, respectively [12]. 

The inherent weakness of the FRCA has been a significant obstacle to extend the use of FRCA to concrete members so that an effective way to improve the mechanical performance has been researched. One possible way to overcome the adverse effect of RCA on its mechanical performance is by employing mineral admixtures. There are three representative mineral admixtures, i.e., fly ash (FA), ground-granulated blast-furnace slag (GGBS), and silica fume (SF). According to the ACI committee about the mineral admixtures in concrete [13,14,15], the natural aggregate concrete gains quite better mechanical performance by using the high-volume mineral admixtures at 28 days or long-term curing days. Additionally, the pozzolanic effect of the mineral admixtures can reduce the hydration heat, which can decrease shrinkage cracks and improve mechanical and durability properties for CRCA concrete [1,4]. Recently, CRCA concrete has been used in self-compacting concrete (SCC) with the mineral admixtures [16,17]. For FRCA concrete, the mixing proportions for better mechanical performance may be more important by using the mineral admixtures, which can reduce the porosity of concrete [18]. 

There is a little previous research on the effect of the mineral admixtures for FRCA concrete. Fly ash addition, over 50% showed strength reduction of 7.5% of normal strength [19] and 60% reduction in high strength [7] at 28 days. GGBS addition, improved the mechanical performance of FRCA concrete up to 30% GGBS addition and the higher addition of 70% showed a 56% decrease of the strength [20]. For SF addition, normal strength concrete presented 4% strength increases [21], while high strength concrete showed a 40% decrease to the strength [22] at 28 days. 

From the literature, it was found that the mechanical performance of FRCA concrete could be reduced in the specific amount of the mineral admixtures in 28 days, while long-term strength can be improved as well. Due to the significant difference in FRCA’s quality, the relationship between the type of the mineral admixtures and the FRCA needs to be further investigated [23]. The lack of studies about FRCA concrete with the mineral admixtures should be extensively investigated, especially for the use of high-volume FRCA concrete members. 

The purpose of this study is to investigate the mechanical performance of concrete with commercially produced FRCA employing the three types of the mineral admixtures, namely FA, GGBS, and SF. The replacement ratios of FRCA are 50 and 100% regarding high volume and complete usage of the FRCA. The amount of the mineral admixtures in FRCA concrete considers two types of dosages; normal dosages by the ACI committee and half-normal dosage. Mechanical performances are investigated using compressive strength, splitting tensile strength, and elastic modulus at 28 days. Additionally, a mercury intrusion porosimetry (MIP) test is employed to investigate the relationship between the mechanical strength and porosity of FRCA concrete using high amounts of the mineral admixtures. 

## 2. Experimental Program 

### 2.1. Materials

**Figure 1:**
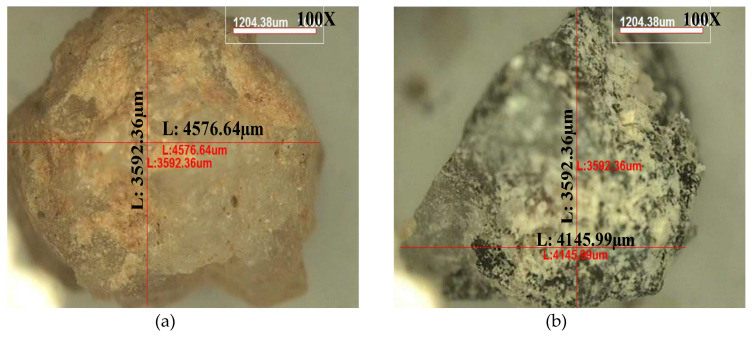
Microscope image of fine aggregates used for this study for (**a**) natural fine aggregate and (**b**) fine recycled concrete aggregate.

The aggregates used for this study are classified as natural coarse aggregate (NCA), natural fine aggregate (NFA), and fine recycled concrete aggregate (FRCA). NCA is prepared by using crushed gravel, and NFA is river sand. FRCA is provided by a domestic manufacturer in Korea (IK Co. Ltd., Incheon, Korea), which has produced good quality of FRCA [24,25,26]. Figure 1 shows the microscope images for NFA and FRCA used for this study. NFA has clean surface and fair roughness, while FRCA has many of the remaining old cement pastes and particles, causing the high porosity in FRCA concrete as compared with NFA concrete. Additionally, the surface is quite rough due to the crushing process of the demolished concrete members and it may affect the workability and mechanical properties.

**Figure 2:**
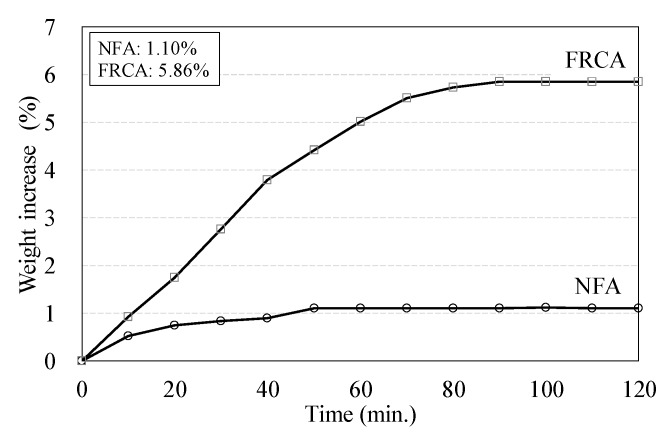
Weight increase of fine aggregates according to time.

The water absorption is a significant issue to determine the quality of the FRCA. The measured water absorption by ASTM C128 (2015) [27] indicated that it is 1.1% for NFA and 5.86% for FRCA, respectively, as shown in Figure 2. It was higher than that of NFA, however, it can be compatible or less than the design specifications, such as RILEM TC 121 (1994) (10%) [5], Korean Construction Specification (2016) (3–4%) [28]. The grain size distributions of the aggregates satisfied the requirements of the standard particle size distribution by ASTM C136 (2014) well [29].

There are four binders, including three types of the mineral admixtures such as fly ash (FA, Sampyo Cement, Seoul, Korea), granulated blast furnace slag (GGBS, Sampyo Cement, Seoul, Korea), and silica fume (SF, Sampyo Cement, Seoul, Korea). The chemical compositions of the binding materials were measured using X-ray fluorescence (XRF, Sampyo Cement, Seoul, Korea), and the specific surface area was evaluated according to ASTM C204 (2011) [30], which are presented in Table 1. These data were obtained from the manufacturer. The mineral admixtures impose the pozzolanic effect on the hardened concrete due to the high fraction of silica ingredients.

### 2.2. Mixing Design

The aggregates are prepared for the saturated surface dry (SSD) condition by ASTM C128 (2015) [27]. Accordingly, the effective water to cement ratio (*W_ef_*/*C*) is the same with the total water to cement ratio (*W_total_*/*C*). The water to binder ratio is 42.5% in volume and the target compressive strength is 27 MPa for the normal strength concrete specified by the standard of recycled aggregate concrete [28]. There are three mixing groups according to the replacement ratio of FRCA, i.e., 0%, 50%, 100%. Each mixing group has seven mixing proportions to the mineral admixtures. The dosages of the admixtures of FA, GGBS, and SF are determined with the normal dosage (30%, 40%, and 5.0%, respectively) based on ACI 232 [13] for fly ash, ACI 233 [14] for GGBS, and ACI 234 [15] for silica fume. The other dosage variable is half-normal dosage (15%, 20%, and 2.5%, respectively) based on the literature reviews which showed the decrease of the mechanical performance of FRCA concrete with increase of the amount of the mineral admixtures. Table 2 summarizes the mixing proportions. 

The slump of FRCA concrete can be influenced by the rough surface so that the slump may decrease with the increase in amount of FRCA. In order to keep the high slump property, the polycarboxylate-based superplasticizer (SP) is used in the mixing design with the dosage of 1% to the amount of cement. This is because of the possible use of the FRCA concrete to the filled concrete to the steel tube. Table 3 presents the measured slump and air content for the test specimens.

The three stage mixing process are employed. It is to mainly overcome the high porous nature of FRCA. First, the SSD coarse and fine aggregates are mixed during the 30 s. Next, the cement and the mineral admixtures are added and mixed for 30 s without water. Lastly, water and chemical admixture are added and mixed during the 90 s and 180 s. This mixing process was motivated by previous studies to improve the ITZ characteristics for recycled aggregates [31].

### 2.3. Specimen Fabrication and Curing 

Six test cylinders with Φ100 × 200 mm were produced for each specimen, hence, a total of 126 cylinders were fabricated for measuring the compressive strength, splitting tensile strength, and elastic modulus. One day after casting, cylinder specimens are demolded and cured for 28 days in an air-dry laboratory. According to the national weather service, the average temperature and the average relative humidity (RH) were 23.1 ± 8.6 °C and 68.7 ± 18.4%, respectively, during the curing period. This was under outdoor conditions so that the curing room condition would be much more stable with less variations on temperature and RH. For the porosity measurement, the FRCA specimens of 50 × 50 × 50 mm^3^ are fabricated from the five mixing proportions of R0, R100, R100FA30, R100GGBS40, and R100SF5.0, respectively.

### 2.4. Test Methods 

The compressive strength test was conducted according to ASTM C39 (2015) [32] at 28 d. The surface of each cylinder specimen was evenly treated using a grinding machine. A load was applied using a universal testing machine (UTM) with the loading rate of 0.3 MPa/s. The splitting tensile strength at 28 days was tested by ASTM C496 (2011) [33]. Two pieces of plywood of size 3 × 25 × 210 mm were placed on each side of the cylinder. A load applied using the UTM with the loading rate of 1.4 MPa/min. The elastic modulus was measured at 28 days by ASTM C469 (2014) [34]. For the porosity measurement, the mercury intrusion porosimetry (MIP) method was employed by ASTM D4404 [35] and the FRCA specimens broken at 28 days were tested to measure the total porosity. 

## 3. Experimental Results

### 3.1. Compressive and Splitting Tensile Strength 

Table 4 is the results of the compressive and splitting tensile strength at 28 d. Strength reduction of R100 concrete was observed up to 12% as compared with R0 concrete. The mineral admixtures improved the strength for R0 and R50 concrete regardless of the types and dosages, while R100 concrete showed strength decrease to the normal dosage of the mineral admixtures. Among the mineral admixtures, SF contributed to the highest increase in strength for R0 concrete, while R50 and R100 concrete did not show a significant improvement of the strength by using SF. The effect of the splitting tensile strength by using the mineral admixtures was similar to that of the compressive strength. However, the use of the mineral admixtures could not give the increase in the tensile strength for R100 concrete as compared with R0 concrete. The ratio of tensile to compressive strength was ranged from 0.11 to 0.13 on average, which was the general range between the compressive and tensile strength [18]. 

It should be noted that the ratio slightly increases with increase in FRCA. This may be caused by the roughness of the FRCA to make better resistance to the tensile failure. Note that the failure mechanism of concrete with the mineral admixtures is affected by the strength of aggregate, the strength of the paste, and ITZ property. The increase in the amount of the FRCA leads to increase the possibility of existence of weak strength of aggregate and the porosity of ITZ on the old cement paste of FRCA. Furthermore, the increase in the amount of the mineral admixtures brings the higher retardation of the hydration of cement paste at 28 d. 

**Figure 3:**
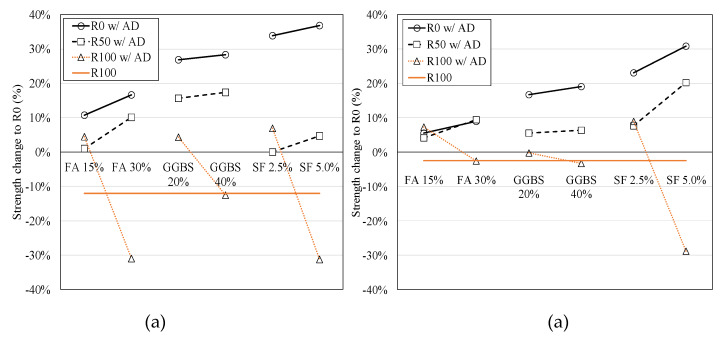
Strength change of test specimens to R0 concrete for (**a**) compressive strength, and (**b**) splitting tensile strength.

Figure 3 presents the strength change according to the amount of FRCA and the dosage of the mineral admixtures. For R0 concrete with natural fine aggregate, silica fume with high fineness and the amount of silica products showed the highest development of the compressive strength among other mineral admixtures. The compressive strength of R0 and R50 concrete increased with the increase of the mineral admixtures, while R100 concrete showed significantly lower strength at the normal dosage. This was because the higher retardation of hydration of cement paste leaded to the weak integrity of material such as ITZ property so that interlocking of aggregate is relatively weak. The splitting tensile strength showed the similar trend with the compressive strength. However, the decrease of the tensile strength of FRCA concrete was not as much as that of the compressive strength. This may be due to the high roughness of FRCA so that it would be good tensile resistance.

### 3.2. Elastic Modulus

**Figure 4:**
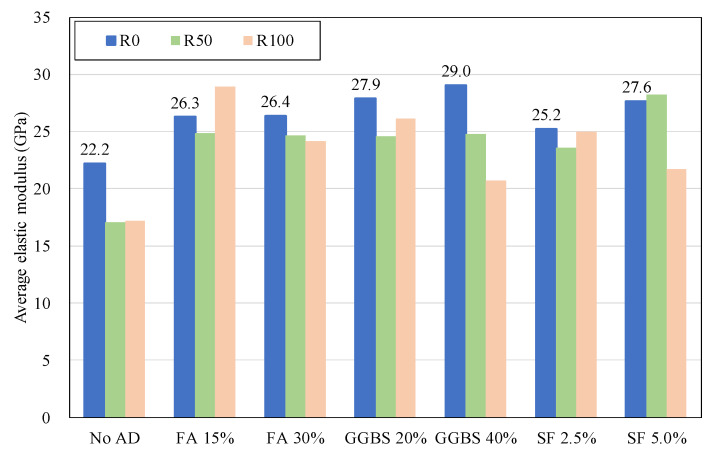
Development of elastic modulus according to the admixtures.

**Figure 5:**
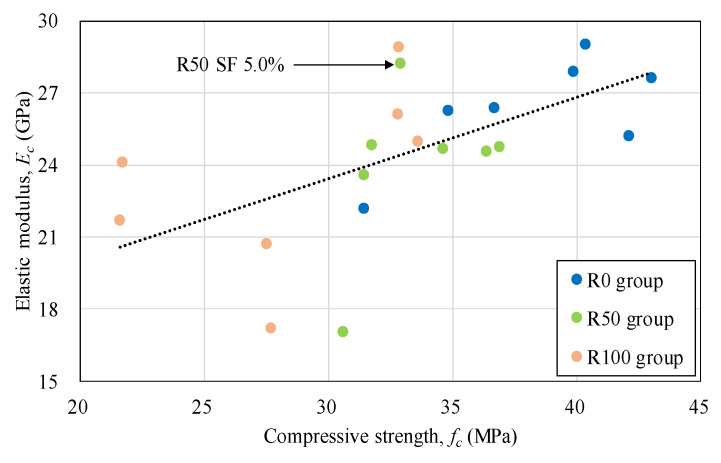
Relationship between compressive strength and elastic modulus.

The addition of the mineral admixtures improved the elastic modulus for R0, R50, and R100 concrete as shown in Figure 4. It was found that the use of the mineral admixtures considerably improved the elastic modulus of FRCA concrete. The half-normal dosage of the admixtures was more effective against R100 concrete for enhanced elastic modulus than R50 concrete. The normal dosage of the admixtures to R100 concrete showed the same decrement trend with the compressive strength although it was still higher than FRCA concrete without the admixtures. Figure 5 displays the relationship between compressive strength and elastic modulus of the test specimens. Although R100 group had larger variation than R0 and R50 group, all specimens exhibited good linear correlation. For R50 SF 5.0% specimen, the average value was somewhat higher than R50 SF 2.5%. This may be caused by measurement error for averaging with only three numbers of cylinders for both the SF 2.5% and 5.0% specimens. 

### 3.3. Porosity

**Figure 6:**
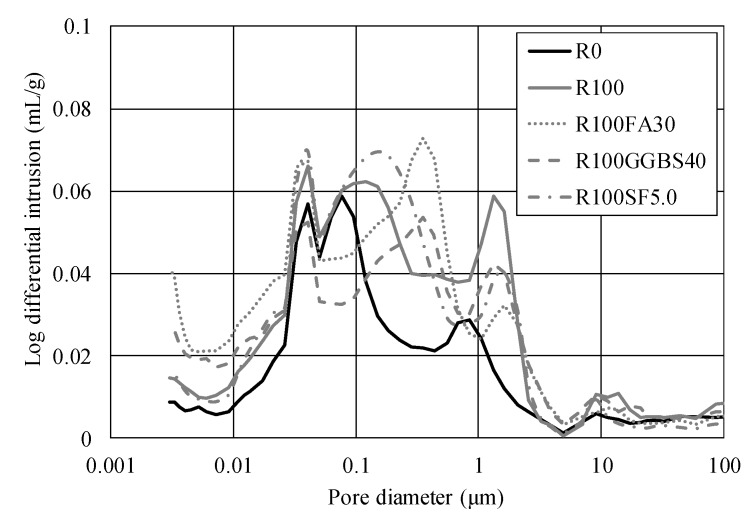
Pore diameter distribution.

The pore size of hardened concrete is an important factor to determine the material property. The common boundary of size of the micro- and macropore is 0.05 μm [36]. Figure 6 shows the result of the porosity measurement by MIP for the FRCA specimens with normal dosages of the mineral admixtures. A micropore distribution smaller than 0.05 μm is differently observed according to the types of the mineral admixtures. Note that the mineral admixtures contribute to generate the micropores which are mainly related to non-mechanical behavior, such as the drying shrinkage or creep. For macropores larger than 0.05 μm, however, they can degrade the compressive strength of concrete or the durability caused by penetration of CI ions. Figure 5 shows that R100 concrete has a relatively high volume of macropores. Table 5 shows the result of the porosity measurement of the test specimens. The R100 specimen exhibited 6.7% higher porosity than R0 specimen so that it can explain the low strength of FRCA concrete. For adding the effect of the mineral admixtures, it slightly reduces the amount of the macropores to R0 specimen, however, it is still higher than the R0 specimen at 28 days. The possible reason may be the retardation of hydration of cement paste caused by the pozzolanic reaction to the normal dosage of the mineral admixtures.

## 4. Discussion 

### 4.1. Relationship Between the Tested and the Predicted Splitting Tensile Strength 

Table 6 exhibits the comparison results of the tested and predicted splitting tensile strength. The predicted values are obtained by using previously published five formulas, including code equations [37,38,39,40,41]. The formulas underestimate the tensile strength of the test specimens, hence, they can give safe prediction results. Admixture additions mostly bring the higher underestimated prediction than that of R0 concrete. This can explain the enhancement effect of splitting tensile strength by using the mineral admixtures. Additionally, FRCA has a rough surface property so that it additionally increases the strength. 

### 4.2. Comparisons of Predicted Elastic Modulus by Design Code Equations 

Table 7 shows the measured elastic modulus and the prediction results by ACI 318-14 [37] and Model Code 2010 [39]. The design code overestimated the measured elastic modulus of concrete. For R50 and R100 concrete, it shows higher overestimated results than R0 concrete. When the mineral admixtures are added, the improved integrity of material property such as ITZ property causes the increase in the elastic modulus, hence, it releases the overestimated prediction by design codes. 

### 4.3. The Effect of the Admixtures on the Strength 

**Figure 7:**
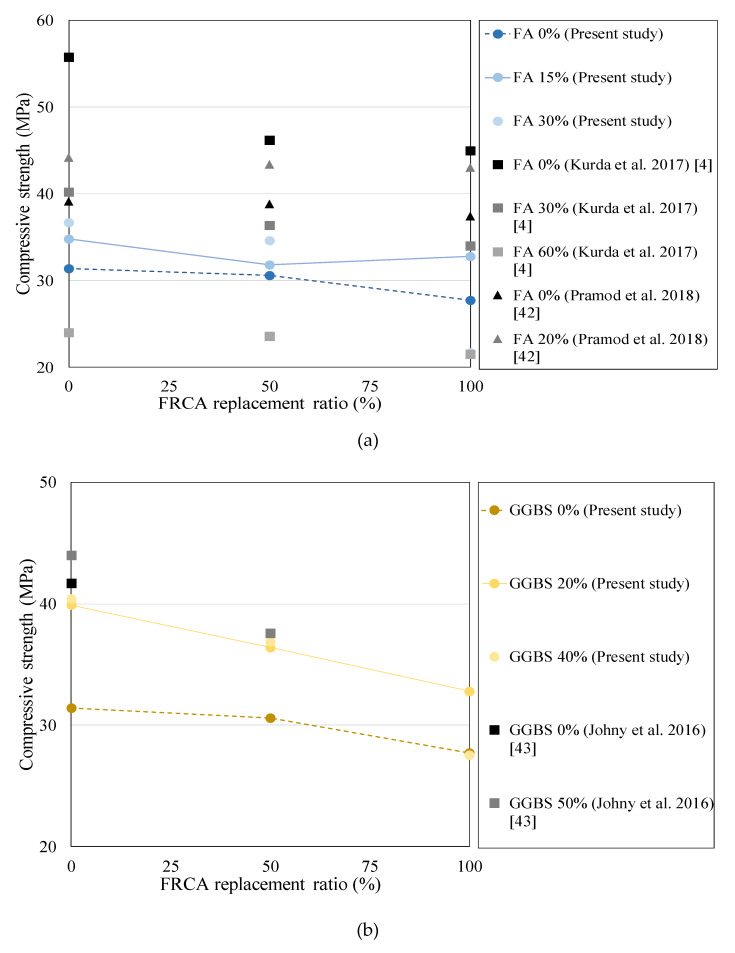

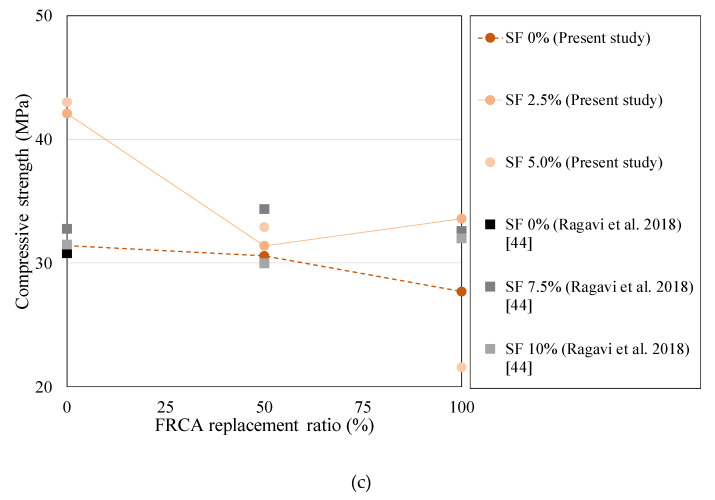
Effect of the admixtures on the strength for (**a**) FA, (**b**) GGBS, and (**c**) SF.

Figure 7 shows the comparative study about the compressive strength of FRCA concrete according to the different mineral admixtures. Note that the dotted line and the solid line represent R0 concrete and the half-normal dosage of the mineral admixtures in the present study, respectively. 

A previous study [7] demonstrated that the high dosage of FA over 30% could degrade the mechanical performance of FRCA concrete. Note that they used the lower quality of FRCA with water absorption of 12% for high strength concrete. The weak ITZ might significantly contribute to the large reduction of strength as compared with NFA concrete, whereas the normal concrete using FRCA with water absorption of 11.5% showed good strength increases to FA 20% [42]. The present study using FA also exhibits good enhancement of strength at FA 15% even for R100 concrete. As a result, 15%–30% dosage of FA is valid for R50 concrete and 15% of that is proper for R100 concrete, which has normal strength and moderate water absorption of 5.86% in this study. 

For the GGBS effect on strength, the literature with water absorption of 3.6% [43], which had very good quality of ITZ of FRCA concrete, was selected for a comparative study. Although they used the quality of the FRCA, FRCA 50% concrete showed quite low strength at GGBS 50% as compared with the NFA concrete. This could explain that the high dosage of GGBS resulted in insufficient integrity on ITZ of FRCA concrete until 28 days. On the other hand, it might exhibit better long-term strength due to the high pozzolanic effect. Based on the present study, both of the GGBS 20% and 40% exhibits significant increase of strength for R0 and R50 concrete. R100 concrete with GGBS 40%, however, has no strength development. This may explain why the normal dosage of GGBS has not been valid for sufficient integrity of the material and the weaker interlocking of aggregates for R100 concrete at 28 days. Although there is the limitation of the number of test results, the effective dosage of GGBS for the mechanical performance may be 20%–40% for R50 concrete and 20% of that for R100 concrete. 

For SF effect on strength, the literature with water absorption of 2.1% [44] which was of higher quality of FRCA was selected for comparative study. The results showed that the SF 7.5% gave the strength increase in R50 and R100 concrete as compared with the NFA concrete. Additionally, SF 10% resulted in a not significant decrease of strength. This could explain that high quality of FRCA closed to NFA could have good material integrity, such as ITZ property and interlocking of aggregates using high pozzolanic admixtures so that it contributed to improve the strength. Note that SF 2.5% and 5.0% contribute the considerable increase of strength for R0 concrete. For moderate quality of FRAC with 5.86% of water absorption used for this study, R50 concrete has the strength development at the normal dosage of 5.0%, while R100 concrete is not allowed to use the SF 5.0%. As a result, the effective dosage of SF may be 5.0% and 2.5% for R50 and R100 concrete, respectively.

### 4.4. Total Porosity Effect on the Strength 

**Figure 8:**
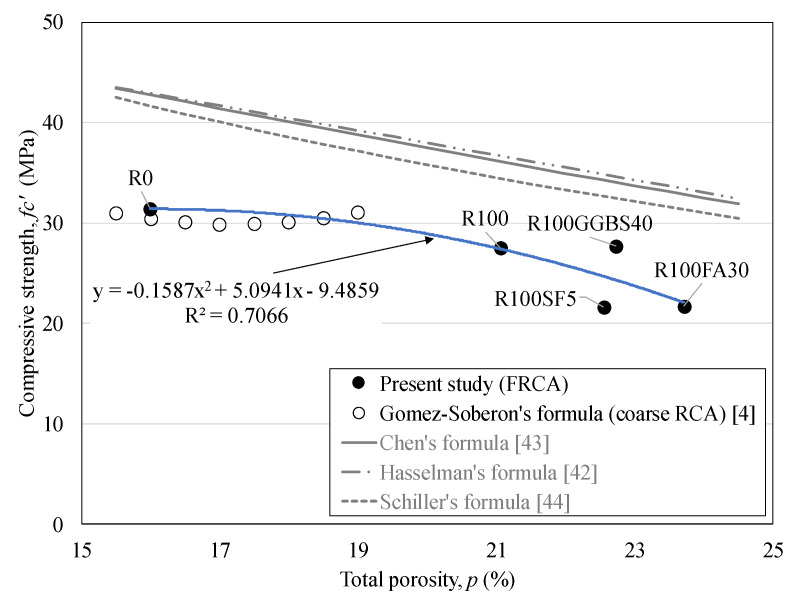
Relationship between the compressive strength and the total porosity.

Increase of porosity in concrete leads to decrease of mechanical property. There are some of the porosity-strength relationship of previous researches [4,45,46,47]. For CRCA concrete, the strength was empirically predicted, according to the total porosity (%). From these literatures, the maximum value of the measured total porosity was at 18%. For FRCA concrete, however, the total porosity must be higher than that of CRCA concrete due to the inherent high porosity of the FRCA. Figure 8 exhibits the comparison results of the relationship between the compressive strength and the total porosity. The prediction formula by previous research [4] could not be available beyond the total porosity of 20%. The other formulas were about cementitious materials using natural aggregates. The prediction trend showed that the compressive strength decreased with the increase of the total porosity. However, there is a large difference in the present test results. This is because of the low development of the strength of R100 concrete from the high porosity although the mineral admixtures are added. 

## 5. Conclusions

The study investigates the effect of the mineral admixtures on the mechanical performance of FRCA concrete, which includes the compressive strength, splitting tensile strength, and elastic modulus. Based on experimental results, the effective dosages of the mineral admixtures are discussed for FRCA concrete with normal strength and moderate water absorption of 5.86%. The key findings of this study are as follows:

1. The mineral admixtures enhance the mechanical performance of FRCA concrete as compared with NFA concrete with no mineral admixtures. For FRCA 50% replaced concrete, every mineral admixture and dosage can bring better mechanical performance. FRCA 100% replaced concrete, however, the half-normal dosages, thus, FA 15%, GGBS 20%, and SF 2.5% are available for enhancing the mechanical performance. 

2. The admixture additions improve the measured elastic modulus of FRCA concrete with about 45%. The strength enhancement of FRCA concrete by the mineral admixtures can be good for improving the predictability of the elastic modulus predicted by the code equations. 

3. The relationship between the total porosity and the compressive strength of the FRCA concrete does not comply with those of cementitious materials using natural aggregates and the CRCA concrete. Due to FRCA concrete has inherently higher porosity than that of CRCA, the strength shows less at the same level of porosity. Extra datasets of the FRCA concrete will be able to be used for developing proper relationship between the strength and the total porosity.

## Figures and Tables

**Table 1:** Chemical compositions of binders used for this study.

Types of Binders	Fineness (cm^2^/g)	Density	Chemical Composition (%)							
			LOI **	SiO_2_	Al_2_O_3_	Fe_2_O_3_	CaO	MgO	SO_3_	K_2_O
OPC *	3616	3.15	2.32	21.62	5.77	3.36	61.43	2.38	2.1	1.02
FA	3520	2.21	4.84	52.09	21.22	6.57	11.49	1.64	1.44	0.71
GGBS	4080	2.92	0.75	34.25	15.14	0.91	39.48	5.96	3.51	0
SF	160,000	2.21	0.38	96.65	1.87	0.03	0	0.19	0.32	0.56

* Ordinary Portland cement, ** loss on ignition.

**Table 2:** Mixing proportions.

Specimens	Admixture Additions		Unit Weight (kg/m^3^)							
	AD * Type	% **	Water	OPC	FA	GGBS	SF	Natural Sand	Recycled Sand (Fine RCA)	Crushed Gravel
R0	No AD		168	395				860		871
R0FA15	FA	15		336	42					
R0FA30		30		277	84					
R0GGBS20	GGBS	20		316		73				
R0GGBS40		40		237		146				
R0SF2.5	SF	2.5		385			7			
R0SF5.0		5.0		376			13			
R50	No AD		168	395				430	398	879
R50FA15	FA	15		336	42					
R50FA30		30		277	84					
R50GGBS20	GGBS	20		316		73				
R50GGBS40		40		237		146				
R50SF2.5	SF	2.5		385			7			
R50SF5.0		5.0		376			13			
R100	No AD		168	395					843	888
R100FA15	FA	15		336	42					
R100FA30		30		277	84					
R100GGBS20	GGBS	20		316		73				
R100GGBS40		40		237		146				
R100SF2.5	SF	2.5		385			7			
R100SF5.0		5.0		376			13			

* AD is the admixture, ** Replacement ratio to the cement in weight.

**Table 3:** Slump and air content.

Specimens	Slump (mm)		Air Content (%)	
	Measured	Avg. (COV) *	Measured	Avg. (COV) *
R0	220	214 ± 3.3 (2%)	4	4 ± 0.2 (6%)
R0FA15	215		3.7	
R0FA30	210		3.5	
R0GGBS20	215		4.1	
R0GGBS40	212		3.9	
R0SF2.5	210		3.6	
R0SF5.0	215		3.5	
R50	220	212 ± 4.4 (2%)	4.9	5 ± 0.3 (6%)
R50FA15	215		4.6	
R50FA30	212		4.2	
R50GGBS20	215		4.9	
R50GGBS40	210		5	
R50SF2.5	210		4.9	
R50SF5.0	205		4.5	
R100	215	212 ± 2.3 (1%)	5.5	5 ± 0.4 (8%)
R100FA15	215		4.8	
R100FA30	210		4.2	
R100GGBS20	212		5.2	
R100GGBS40	210		5	
R100SF2.5	210		5.3	
R100SF5.0	215		5	

* The average slump or Air content ± Standard deviation (Coefficient of variation %).

**Table 4:** Compressive and tensile strength results.

Specimens	Compressive Strength *f_c_* (MPa)		Δ_fc_ (%)	Splitting Tensile Strength *f_st_* (MPa)		Δ_fst_ (%)	*f_st_/f_c_*
	Measured	Avg. *		Measured	Avg. *		
R0	31.2, 32.1, 31.0	31.4 ± 0.5	0%	3.68, 3.72	3.70 ± 0.02	0%	0.12
R0FA15	34.6, 35.0	34.8 ± 0.2	10.7%	3.82, 4.20, 3.70	3.91 ± 0.21	1.4%	0.11
R0FA30	36.9, 35.7, 37.4	36.7 ± 0.7	16.6%	4.23, 4.00, 3.86	4.03 ± 0.15	4.6%	0.11
R0GGBS20	43.6, 41.9, 34.1	39.9 ± 4.1	26.8%	4.37, 4.27	4.32 ± 0.05	15.5%	0.11
R0GGBS40	41.2, 39.5	40.4 ± 0.9	28.4%	4.59, 4.23, 4.41	4.41 ± 0.05	14.4%	0.11
R0SF2.5	39.7, 44.3, 42.3	42.1 ± 1.9	33.9%	4.17, 4.51, 4.98	4.56 ± 0.33	18.2%	0.11
R0SF5.0	40.5, 46.0, 42.5	43.0 ± 2.3	36.8%	5.25, 4.68, 4.60	4.84 ± 0.29	25.7%	0.11
R50	28.0, 31.0, 32.7	30.6 ± 1.9	–2.8%	3.78, 3.63	3.71 ± 0.08	–3.8%	0.12
R50FA15	29.9, 33.6	31.8 ± 1.9	1.0%	3.83, 3.69, 4.04	3.85 ± 0.15	0.0%	0.12
R50FA30	35.4, 33.8	34.6 ± 0.8	10.1%	3.94, 4.16	4.05 ± 0.11	5.1%	0.12
R50GGBS20	33.8, 37.7, 37.6	36.4 ± 1.8	15.7%	3.91, 3.90	3.90 ± 0.01	5.5%	0.11
R50GGBS40	37.5, 36.0, 37.2	36.9 ± 0.6	17.4%	3.95, 3.92	3.94 ± 0.02	0.0%	0.11
R50SF2.5	31.8, 32.1, 30.4	31.4 ± 0.7	0.0%	3.90, 3.80, 4.25	3.98 ± 0.19	3.4%	0.13
R50SF5.0	32.7, 33.1	32.9 ± 0.2	4.7%	4.54, 4.36	4.45 ± 0.09	20.3%	0.13
R100	29.0, 26.6, 27.4	27.7 ± 1.0	–12.0%	3.57, 3.66, 3.60	3.61 ± 0.04	–6.3%	0.13
R100FA15	32.7, 33.6, 32.2	32.8 ± 0.6	4.5%	4.26, 4.04, 3.61	3.97 ± 0.27	3.0%	0.12
R100FA30	22.2, 21.2	21.7 ± 0.5	–31.0%	3.63, 3.58	3.60 ± 0.02	–6.5%	0.14
R100GGBS20	31.1, 34.6, 32.7	32.8 ± 1.4	4.3%	3.42, 3.91, 3.75	3.70 ± 0.2	–4.1%	0.11
R100GGBS40	28.4, 27.2, 26.9	27.5 ± 0.6	–12.5%	3.73, 3.43	3.58 ± 0.15	–7.2%	0.13
R100SF2.5	33.7, 33.5	33.6 ± 0.1	6.9%	3.85, 4.16, 4.09	4.03 ± 0.13	2.7%	0.12
R100SF5.0	21.3, 22.2, 21.3	21.6 ± 0.4	–31.3%	2.46, 2.72, 2.73	2.64 ± 0.13	–31.6%	0.12

* The average ± standard deviation.

**Table 5:** Result of porosity measurement.

Types of Meaxurement	R0	R100	R100FA30	R100GGBS40	R100SF5.0
Total intrusion vol. (mL/g)	0.0803	0.1254	0.1303	0.1136	0.1203
Total pore area (m^2^/g)	7.047	10.492	17.423	13.673	10.719
Average pore diameter (µm)	0.0456	0.0478	0.0299	0.0332	0.0449
Total porosity (%)	15.99	22.73	23.71	21.06	22.55

**Table 6:** Tensile strength prediction by previous formulas.

Specimens	Tensile Strength, *f_st_* (MPa)	Tensile Strength Prediction (MPa)				
		ACI318-14 [37]	Model Code 2010 [39]	Raphael [40]	Carino and Lew [38]	Zain et al. [41]
R0	3.70 ± 0.02	3.14 (1.18*)	3.39 (1.09)	3.12 (1.19)	3.15 (1.18)	3.07 (1.21)
R0FA15	3.91 ± 0.21	3.30 (1.18)	3.53 (1.11)	3.34 (1.17)	3.38 (1.16)	3.29 (1.19)
R0FA30	4.03 ± 0.15	3.39 (1.19)	3.60 (1.12)	3.46 (1.17)	3.51 (1.15)	3.40 (1.18)
R0GGBS20	4.45 ± 0.05	3.54 (1.26)	3.72 (1.20)	3.66 (1.22)	3.72 (1.20)	3.59 (1.24)
R0GGBS40	4.41 ± 0.15	3.56 (1.24)	3.74 (1.18)	3.69 (1.20)	3.76 (1.17)	3.62 (1.22)
R0SF2.5	4.56 ± 0.33	3.63 (1.25)	3.80 (1.20)	3.79 (1.20)	3.87 (1.18)	3.72 (1.23)
R0SF5.0	4.84 ± 0.29	3.67 (1.32)	3.83 (1.26)	3.85 (1.26)	3.93 (1.23)	3.77 (1.29)
R50	3.71 ± 0.08	3.10 (1.20)	3.35 (1.11)	3.06 (1.21)	3.08 (1.20)	3.01 (1.23)
R50FA15	3.85 ± 0.15	3.16 (1.22)	3.40 (1.13)	3.14 (1.23)	3.17 (1.22)	3.09 (1.25)
R50FA30	4.05 ± 0.11	3.29 (1.23)	3.52 (1.15)	3.33 (1.22)	3.37 (1.20)	3.27 (1.24)
R50GGBS20	3.90 ± 0.01	3.38 (1.15)	3.59 (1.09)	3.44 (1.13)	3.49 (1.12)	3.38 (1.15)
R50GGBS40	3.94 ± 0.02	3.40 (1.16)	3.61 (0.19)	3.47 (1.13)	3.52 (1.12)	3.42 (1.15)
R50SF2.5	3.98 ± 0.19	3.14 (1.27)	3.39 (1.18)	3.12 (1.28)	3.15 (1.27)	3.07 (1.30)
R50SF5.0	4.45 ± 0.09	3.29 (1.35)	3.52 (1.27)	3.32 (1.34)	3.36 (1.32)	3.27 (1.36)
R100	3.61 ± 0.04	2.95 (1.23)	3.22 (1.12)	2.87 (1.26)	2.87 (1.26)	2.80 (1.29)
R100FA15	3.97 ± 0.27	3.21 (1.24)	3.45 (1.15)	3.21 (1.24)	3.24 (1.22)	3.16 (1.26)
R100FA30	3.60 ± 0.02	2.80 (1.29)	3.09 (1.17)	2.68 (1.35)	2.67 (1.35)	2.60 (1.39)
R100GGBS20	3.70 ± 0.2	3.21 (1.15)	3.45 (1.07)	3.21 (1.15)	3.24 (1.14)	3.16 (1.17)
R100GGBS40	3.58 ± 0.15	2.94 (1.22)	3.21 (1.11)	2.85 (1.25)	2.86 (1.25)	2.79 (1.28)
R100SF2.5	4.03 ± 0.13	3.25 (1.24)	3.48 (1.16)	3.26 (1.24)	3.30 (1.22)	3.21 (1.26)
R100SF5.0	2.64 ± 0.13	2.60 (1.01)	2.92 (0.90)	2.43 (1.09)	2.41 (1.09)	2.33 (1.13)

* The average ratio of the measured *f_st_* to the predicted (the five formulas) for splitting tensile strength.

**Table 7:** Predicted elastic modulus by the design codes.

Specimens	Avg. * (GPa)			Measured/ACI318 [37]	Measured/Model Code 2010 [39]
	Measured	ACI 318 [37]	Model Code 2010 [39]		
R0	22.2 ± 0.0	26.3 ± 1.26	31.5 ± 1.01	0.84	0.71
R0FA15	26.3 ± 1.44	27.7 ± 0.08	21.7±0.06	0.95	0.81
R0FA30	26.4 ± 2.19	28.5 ± 0.28	33.2 ± 0.22	0.93	0.80
R0GGBS20	27.9 ± 1.41	29.6 ± 1.57	34.0 ± 1.21	0.94	0.82
R0GGBS40	29.0 ± 0.0	29.9 ± 0.31	22.8 ± 0.24	0.97	0.85
R0SF2.5	25.2 ± 1.85	30.5 ± 0.68	34.7 ± 0.52	0.83	0.73
R0SF5.0	27.6 ± 0.84	30.8 ± 0.81	35.0 ± 0.61	0.90	0.79
R50	17.1 ± 1.99	26.0 ± 0.83	31.2 ± 0.67	0.66	0.55
R50FA15	24.8 ± 3.95	26.5 ± 0.77	31.6 ± 0.61	0.94	0.79
R50FA30	24.7 ± 1.66	27.6 ± 0.32	32.5 ± 0.25	0.89	0.76
R50GGBS20	24.6 ± 4.06	28.3 ± 0.71	33.1 ± 0.56	0.87	0.74
R50GGBS40	24.8 ± 1.27	28.5 ± 0.25	33.2 ± 0.20	0.87	0.75
R50SF2.5	23.6 ± 0.0	26.3 ± 0.31	31.5 ± 0.25	0.90	0.75
R50SF5.0	28.2 ± 1.47	26.9 ± 0.1	32.0 ± 0.06	1.05	0.88
R100	17.2 ± 1.4	24.7 ± 0.44	30.2 ± 0.36	0.70	0.57
R100FA15	28.9 ± 4.35	26.9 ± 0.24	32.0 ± 0.19	1.07	0.90
R100FA30	24.1 ± 1.81	23.4 ± 2.14	18.6 ± 0.21	1.03	0.87
R100GGBS20	26.1 ± 1.03	26.9 ± 0.59	31.9 ± 0.46	0.97	0.82
R100GGBS40	20.7 ± 1.36	24.6 ± 0.29	30.1 ± 0.24	0.84	0.69
R100SF2.5	25.0 ± 0.83	27.2 ± 0.04	21.5 ± 0.03	0.92	0.78
R100SF5.0	21.7 ± 0.0	21.8 ± 0.21	27.8 ± 0.18	0.99	0.78

* The average ± standard deviation.
